# Ambulatory Blood Pressure Monitoring in Individuals with HIV: A Systematic Review and Meta-Analysis

**DOI:** 10.1371/journal.pone.0148920

**Published:** 2016-02-16

**Authors:** Shia T. Kent, Samantha G. Bromfield, Greer A. Burkholder, Louise Falzon, Suzanne Oparil, Edgar T. Overton, Michael J. Mugavero, Joseph E. Schwartz, Daichi Shimbo, Paul Muntner

**Affiliations:** 1 Department of Epidemiology, University of Alabama at Birmingham, Birmingham, Alabama, United States of America; 2 Division of Infectious Diseases, Department of Medicine, University of Alabama at Birmingham, Birmingham, Alabama, United States of America; 3 Center for Behavioral Cardiovascular Health, Department of Medicine, Columbia University Medical Center, New York, New York, United States of America; 4 Department of Medicine, Vascular Biology and Hypertension Program, University of Alabama at Birmingham, Birmingham, Alabama, United States of America; 5 Applied Behavioral Medicine Research Institute, Department of Psychiatry, Stony Brook University School of Medicine, Stony Brook, New York, United States of America; Azienda ospedaliero-universitaria di Perugia, ITALY

## Abstract

**Introduction:**

Abnormal diurnal blood pressure (BP) rhythms may contribute to the high cardiovascular disease risk in HIV-positive (HIV^+^) individuals. To synthesize the current literature on ambulatory BP monitoring (ABPM) in HIV^+^ individuals, a systematic literature review and meta-analysis were performed.

**Methods:**

Medical databases were searched through November 11, 2015 for studies that reported ABPM results in HIV^+^ individuals. Data were extracted by 2 reviewers and pooled differences between HIV^+^ and HIV-negative (HIV^-^) individuals in clinic BP and ABPM measures were calculated using random-effects inverse variance weighted models.

**Results:**

Of 597 abstracts reviewed, 8 studies with HIV^+^ cohorts met the inclusion criteria. The 420 HIV^+^ and 714 HIV^-^ individuals in 7 studies with HIV^-^ comparison groups were pooled for analyses. The pooled absolute nocturnal systolic and diastolic BP declines were 3.16% (95% confidence interval [CI]: 1.13%, 5.20%) and 2.92% (95% CI: 1.64%, 4.19%) less, respectively, in HIV^+^ versus HIV^-^ individuals. The pooled odds ratio for non-dipping systolic BP (nocturnal systolic BP decline <10%) in HIV^+^ versus HIV^-^ individuals was 2.72 (95% CI: 1.92, 3.85). Differences in mean clinic, 24-hour, daytime, or nighttime BP were not statistically significant. I^2^ and heterogeneity chi-squared statistics indicated the presence of high heterogeneity for all outcomes except percent DBP dipping and non-dipping SBP pattern.

**Conclusions:**

An abnormal diurnal BP pattern may be more common among HIV^+^ versus HIV^-^ individuals. However, results were heterogeneous for most BP measures, suggesting more research in this area is needed.

## Introduction

With the introduction of effective antiretroviral therapy (ART), HIV-positive (HIV^+^) individuals are living longer and care now includes the management of chronic comorbidities, including cardiovascular disease (CVD) risk factors [1]. Clinic hypertension, defined as clinic-measured systolic blood pressure (SBP) ≥ 140 mm Hg or diastolic blood pressure (DBP) ≥ 90 mm Hg or use of antihypertensive medication, is a major risk factor for CVD in the general population and also in HIV^+^ individuals [1, 2]. HIV^+^ individuals have increased CVD risk compared to HIV-negative (HIV^-^) individuals, even after accounting for blood pressure (BP) measured in the clinic [1–3].

HIV infection and HIV-induced proteins directly disrupt the body’s circadian clock [4]. The autonomic dysfunction and abnormal baroreflex function found in HIV^+^ individuals are also associated with circadian rhythm abnormalities [5–7]. HIV^+^ individuals experience high levels of social stigma, psychological stress, and pain, which are associated with sleep disturbances [8–10]. Ambulatory BP monitoring (ABPM) complements clinic BP by quantifying diurnal BP patterns including elevated nighttime BP and reduced nocturnal BP dipping [11]. These measures have been associated with CVD risk in the general population, independent of clinic BP [11, 12]. Given that HIV^+^ status is associated with an abnormal circadian rhythms, these individuals may have a greater risk of elevated nighttime BP and blunted nocturnal BP dipping, compared to HIV^-^ individuals. ABPM can also assess 24-hour BP variability, and phenotypes defined by discordance between clinic and out-of-clinic hypertension status, including white coat hypertension (i.e., the presence of hypertension based on clinic BP measurements with non-elevated out-of-clinic BP) and masked hypertension (i.e., the absence of hypertension based on clinic BP measurements with elevated out-of-clinic BP) [11, 13, 14]. Increased 24-hour BP variability and masked hypertension are associated with increased CVD risk whereas the association between white coat hypertension and CVD risk is less clear [11, 15].

A higher prevalence of abnormal ABPM phenotypes including elevated nighttime BP, reduced nocturnal BP dipping, increased 24-hour BP variability, masked hypertension among HIV^+^ individuals, compared with HIV^-^ individuals, may provide insight into the mechanisms underlying their elevated CVD risk [11, 13, 16]. Therefore, we conducted a systematic review of studies that reported results of ABPM in HIV^+^ individuals, and then performed meta-analyses of the published data comparing ABPM measures for HIV^+^ and HIV^-^ individuals.

## Methods

Two investigators (STK and SGB) independently assessed articles for eligibility, extracted relevant data, and assessed the eligible articles for bias, with a third investigator (PM) consulted if needed.

### Search Strategy and Selection Criteria

Studies were included if they met the following criteria: (1) recorded ABPM for ≥ 24 hours, (2) enrolled HIV^+^ individuals, and (3) if HIV^-^ individuals were included, ABPM measures were reported for HIV^+^ individuals separately. We excluded studies that were only reported in letters to the editor, commentaries, meeting abstracts, editorials, or review articles. The following databases were searched through November 11, 2015: MEDLINE, Database of Abstracts of Reviews of Effects, Health Technology Assessment Database, Scopus, Cumulative Index to Nursing and Allied Health Literature, ProQuest Dissertations & Theses, and ClinicalTrials.gov. The MEDLINE search strategy is provided in the S1 Appendix. Terms for the other databases were adapted as appropriate. To supplement the database term searches, a PubMed “related articles” search was conducted. This search was performed using articles that were identified for abstraction from the initial results. We also performed a manual search of the reference lists from the abstracted articles and review articles produced by the searches. The title and abstract of identified articles were reviewed, and those deemed ineligible were excluded. The full text for the remaining articles were retrieved and reviewed for eligibility.

### Data Extraction

We abstracted study characteristics (country of origin, inclusion/exclusion criteria, and sample size) from eligible studies. We also abstracted participant characteristics, use of antihypertensive medication, use of ART, and BP measures reported for HIV^+^ and HIV^-^ participants, separately. When relevant information was not reported, two attempts were made to contact the corresponding author via e-mail to retrieve the study data.

### Study Risk of Bias Assessment

The risk of bias in eligible studies was assessed using *A Cochrane Risk Of Bias Assessment Tool*: *for Non-Randomized Studies of Interventions* (ACROBAT-NRSI) [17]. Using this tool, eligible studies were each compared to an ideal hypothetical study with HIV infection as the randomized exposure and BP measures as outcomes. Antihypertensive medication was considered to be a “co-intervention” leading to potential bias, since its usage may be associated with both HIV status and BP. Overall study risk of bias judgement is reported as low, moderate, serious, or critical.

### Statistical Analyses

Meta-analyses were conducted among all studies with both HIV^+^ and HIV^-^ individuals. We examined the pooled associations of HIV status with clinic SBP and DBP and with ABPM-measured mean 24-hour SBP and DBP, mean daytime SBP and DBP, mean nighttime SBP and DBP, percentage nocturnal SBP and DBP declines, and non-dipping SBP pattern. For each study, the absolute differences in continuous BP phenotypes and 95% confidence intervals (CIs) were calculated between HIV^+^ and HIV^-^ individuals. We also calculated odds ratios (ORs) and 95% CIs for the association between HIV^+^ status and a non-dipping SBP pattern. To obtain overall effect estimates, pooled weighted mean differences for continuous BP measures, ORs for non-dipping SBP pattern, and 95% CIs for all measures were calculated from inverse-variance weighted random-effects models. Heterogeneity was examined using heterogeneity I^2^ and chi-squared statistics. In sensitivity analyses, all meta-analysis models were re-calculated in a leave-one-out method by individually removing each included study, one at a time, to examine whether pooled estimates and heterogeneity statistics changed. Data management and analyses were conducted using SAS 9.3 (SAS Institute, Cary, NC) and Stata 13.1 (Stata Inc., College Station, TX).

## Results

**Fig 1:**
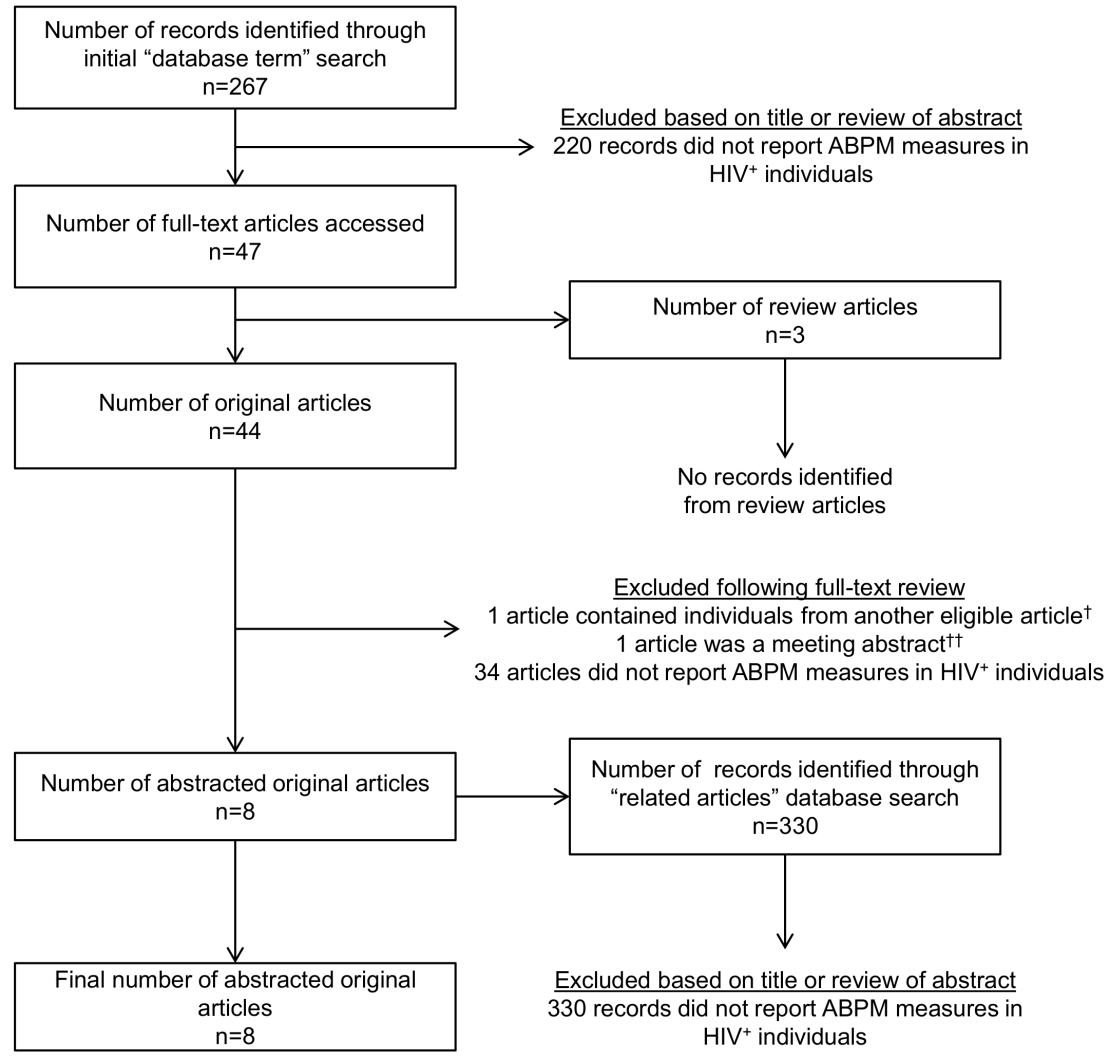
Flow of information for the systematic literature review of ambulatory blood pressure monitoring (ABPM) in HIV^+^ individuals. ^†^Manner, 2010 was not eligible because it consisted of the same 77 HIV^+^ individuals as in Baekken, 2009 but did not include an HIV^-^ comparison group. Measures reported only in Manner, 2010 are included in S1 Table. ^††^Nurenburg, 2015 was not eligible because it was an abstract reported in a supplemental issue of the Journal of Hypertension as a handbook for the 2015 European Society for Hypertension meeting.

The initial database search identified 267 records, of which 220 were excluded because the titles and abstracts indicated that the articles did not report ABPM measures in HIV^+^ individuals (Fig 1). Of the 47 remaining articles for which full-texts were retrieved, 3 were review articles, 1 was excluded because it reported ABPM measures from HIV^+^ individuals already included in a separate eligible article but did not have HIV^-^ controls [18], 1 was excluded because it reported ABPM measures in HIV^+^ individuals in a meeting abstract without a full-text article [19], and 34 records were excluded for not reporting ABPM measures separately in HIV^+^ individuals. Using the remaining 8 articles, supplemental “related articles” searches produced 330 additional records for review. All 330 of these records were excluded because the titles and abstracts indicated that the articles did not report ABPM measures in HIV^+^ individuals. In total, of the 597 records retrieved and reviewed from the original and supplementary searches, 8 studies met the inclusion criteria for abstraction [20–27].

### Study Characteristics

Of the 8 eligible studies, 7 were conducted in Europe [20, 22–27] and 1 in South Africa (Table 1) [21]. All studies were conducted through university-based hospitals or clinics; 6 studies were conducted in a single center [20–22, 24, 26, 27] and 2 studies were conducted in multi-center collaborations [23, 25]. Seven studies reported results from cross-sectional observational data [20, 22–27] and 1 study reported the results from data collected as part of a prospective single-arm 6 month ART trial [21]. The 8 eligible studies were published between 2008 and 2014. The number of HIV^+^ individuals in each study ranged from 40 to 100. The mean age of individuals in each study ranged from 31 to 51 years. Of the 8 studies, 4 were restricted to people with hypertension [20, 25–27], 2 were restricted to individuals without hypertension [21, 23], 1 included equal numbers of patients with and without hypertension [24], and 1 did not report the prevalence of hypertension [22]. All studies excluded individuals taking antihypertensive medication. In 6 of the 8 studies, at least 80% of the HIV^+^ individuals were taking ART [20, 22, 24–27] and 1 study was restricted to individuals not taking ART. For the remaining study, which reported ABPM measures in individuals before and after 6 months of ART, cross-sectional baseline data of ART-naïve individuals were abstracted and used for the current meta-analyses [21].

**Table 1:** Characteristics of studies reporting ambulatory blood pressure monitoring measures in HIV^+^ individuals.

		Sample Size, n			Age, mean years (SD)		Male, %			HIV^+^ on antiretroviral treatment, %	Variables used for matching HIV^+^ and HIV^-^
Year	First Author	HIV^+^	HIV^-^	Country	HIV^+^	HIV^-^	HIV^+^	HIV^-^	Clinic hypertension, %		
2008	Pozdíšek	40	40	Czech Republic	42 (11)	43 (11)	30%	30%	NR	100%	Age, gender
2009	Baekken^b^	77	76	Norway	51 (10)	48 (9)	84%	71%	100%	83%	Not matched
2010	De Socio^c^	52	156	Italy	39 (11)	39 (11)	85%	85%	0%	0%	Age, gender, clinic SBP
2011	Bernardino	43	0	Spain	43 (7)^a^	None	14%^a^	None	100%	81%^a^	No HIV^-^ controls
2012	Grandi	60	60	Italy	NR^d^	NR^d^	87%	87%	50%^d^	100%	Age, gender, BMI, smoking, 24-hour SBP and DBP^e^
2013	Maggi	61	40	Italy	47 (7)	46 (9)	69%	68%	100%	98%	Age, gender, clinic BP^f^
2013	Schillaci	100	325	Italy	48 (9)^a^	48 (10)^a^	72%^a^	69%^a^	100%	92%	Not matched
2014	Borkum^g^	30	17	South Africa	32 (8)	31 (9)	37%	40%	0%	0%	Age, BMI, socio-economic status

Abbreviations: BMI = Body mass index; DBP = diastolic blood pressure; NR = Not reported; SBP = Systolic blood pressure; SD = Standard deviation

For all studies, ambulatory BP procedures were only performed among individuals not taking antihypertensive medication.

All studies consisted of patients recruited from clinical settings. Schillaci, 2013 and De Socio, 2010 also included hospital staff and referrals among HIV^-^ controls.

^a^Characteristics obtained through manuscript author correspondence.

^b^Manner, 2010 is not reported in this table because it included the same 77 HIV^+^ individuals in Baekken, 2009 but did not include an HIV^-^ comparison group.

^c^De Socio, 2010 consisted of individuals in the HIV Exposure and Risk of Metabolic Syndrome (HERMES) cohort.

^d^Grandi, 2012 HIV^+^ and HIV^-^ samples were each 50% hypertensive. This study reported characteristics for HIV^+^ and HIV^-^ each stratified by hypertension status. The mean age (SD) of HIV^+^ and hypertensive individuals was 45 (7) years, of HIV+ and normotensive individuals was 44 (8) years, of HIV^-^ and hypertensive individuals was 45 (6) years, and of HIV^-^ and normotensive individuals was 44 (7) years.

^e^Grandi, 2012 HIV^+^ and HIV^-^ individuals were matched on 24-hour SBP and DBP only within hypertensive and non-hypertensive strata.

^f^Maggi, 2013 notes HIV+ and HIV^-^ individuals were matched on clinic BP, but does not specify SBP or DBP.

^g^Borkum, 2014 reported measures from 30 HIV^+^ individuals at baseline before initiating antiretroviral treatment at baseline and subset of 28 HIV^+^ individuals adherent to antiretroviral treatment for 6 months. This table reports data from the 30 HIV^+^ individuals pre-antiretroviral treatment.

Seven of the 8 studies included an HIV^-^ comparison group [20–26]. Of these 7 studies, 2 did not match HIV^-^ and HIV^+^ individuals on any variables [20, 25]. The remaining 5 studies matched HIV^-^ and HIV^+^ individuals on varying combinations of age, gender, body mass index, smoking, socio-economic status, clinic BP, and mean 24-hour BP [21–24, 26].

### Study Risk of Bias Assessment

Since all 7 studies with HIV^+^ and HIV^-^ individuals excluded participants taking antihypertensive medication, the risk of bias due to potential co-interventions was low. Two studies were assigned a moderate overall risk of bias: Baekken, 2009 which had significantly different (p<0.05) age, gender, and racial distributions by HIV status [20] and Schillaci, 2013 which had significantly different BMI and smoking distributions by HIV status and used two different ABPM device models but did not specify whether they were used in similar proportions among HIV^+^ and HIV^-^ populations [25]. The remaining 5 assessed studies were assigned a low risk of bias [21–24, 26].

### Blood Pressure Measures

Among HIV^+^ individuals, mean clinic SBP ranged from 115 to 149 mm Hg and mean clinic DBP ranged from 76 to 96 mm Hg (Table 2). All studies that reported nocturnal BP declines defined them as 100%* [(daytime BP minus nighttime BP) divided by daytime BP]. Among HIV^+^ individuals, mean nocturnal SBP decline ranged from 5% to 13% and mean nocturnal DBP decline ranged from 11% to 16% (Table 3). All studies that reported a non-dipping SBP pattern defined it as a nocturnal SBP decline <10%. The prevalence of non-dipping SBP pattern ranged from 29% to 82% among HIV^+^ individuals and from 15% to 53% among their HIV^-^ counterparts. The prevalence of non-dipping DBP pattern was not reported in any studies. Other ABPM measures were reported in one or two studies (S1 Table). One study consisting of hypertensive HIV^+^ individuals reported that 81% of individuals had nighttime hypertension, defined as nighttime SBP ≥ 120 mm Hg or DBP ≥ 70 mm Hg. Two studies reported the prevalence of white coat hypertension. Both studies defined clinic hypertension as SBP ≥ 140 or DBP ≥ 90 mmHg. In the first study, which defined non-elevated out-of-clinic BP as daytime SBP/DBP < 130/85 mm Hg, the prevalence of white coat hypertension was 26% among HIV^+^ individuals. The second study defined non-elevated out-of-clinic BP as daytime SBP/DBP < 135/85 mm Hg and reported a white coat hypertension prevalence of 40%. No studies reported the prevalence of masked hypertension or 24-hour BP variability.

**Table 2:** Mean clinic and 24-hour blood pressure values in studies of HIV^+^ individuals.

		Mean clinic BP, mm Hg (SD)		Mean 24-hour BP, mm Hg (SD)	
Year	First Author	SBP	DBP	SBP	DBP
2008	Pozdišek				
HIV^+^		NR	NR	119 (9)	77 (6)
HIV^-^		NR	NR	124 (9)	72 (7)
2009	Baekken				
HIV^+^		149 (12)	91 (8)	135 (16)	84 (11)
HIV^-^		162 (13)	104 (6)	140 (13)	92 (7)
2010	De Socio				
HIV^+^		125 (11)	78 (10)	119 (8)^a^	76 (9)^a^
HIV^-^		125 (10)	78 (9)	120 (11)^a^	75 (6)^a^
2011	Bernardino				
HIV^+^		148 (11)^a^	91 (8)^a^	132 (14)^a^	84 (11)^a^
2012	Grandi^b^				
HIV^+^/hypertensive		141 (14)	96 (8)	135 (11)	88 (8)
HIV^-^/hypertensive		142 (17)	94 (9)	134 (10)	87 (8)
HIV^+^/normotensive		125 (13)	82 (12)	116 (7)	72 (6)
HIV^-^/normotensive		124 (1)	81 (13)	117 (6)	71 (5)
2013	Maggi				
HIV^+^		147 (7)	95 (5)	NR	NR
HIV^-^		145 (7)	93 (4)	NR	NR
2013	Schillaci				
HIV^+^		142 (12)	91 (8)	131 (14)	81 (10)
HIV^-^		141 (11)	90 (8)	126 (10)	81 (8)
2014	Borkum				
HIV^+^ (pre-ART)^c^		115 (10)^a^	76 (8)^a^	114 (26)^a^	73 (13)^a^
HIV^+^ (6 months ART)^c^		117 (16)^a^	76 (15)^a^	114 (16)^a^	73 (15)^a^
HIV^-^		114 (7)^a^	75 (7)^a^	110 (12)^a^	69 (12)^a^

Abbreviations: ART = Antiretroviral therapy; BP = Blood pressure; DBP = Diastolic blood pressure; NR = not reported; SBP = Systolic blood pressure

^a^Measures obtained through manuscript author correspondence

^b^Grandi, 2012 HIV^-^ patients were matched to HIV^+^ patients by 24-hr SBP and DBP

^c^Borkum, 2014 were reported in 30 HIV^+^ individuals at baseline before initiating ART at baseline and subset of 28 HIV+ individuals adherent to ART for 6 months.

**Table 3:** Daytime and nighttime blood pressure, nocturnal blood pressure decline, and non-dipping blood pressure pattern in studies of ambulatory BP among HIV^+^ individuals.

		Mean daytime BP, mm Hg (SD)		Mean nighttime BP, mm Hg (SD)		Mean nocturnal BP decline, % (SD)		Non-dipping BP pattern, %
Year	First Author	SBP	DBP	SBP	DBP	SBP	DBP	
2008	Pozdišek							
HIV^+^		NR	NR	NR	NR	NR	NR	NR
HIV^-^		NR	NR	NR	NR	NR	NR	NR
2009	Baekken							
HIV^+^		139 (17)	88 (11)	128 (17)	78 (11)	8 (7)	12 (8)	60
HIV^-^		147 (14)	96 (7)	129 (14)	83 (8)	13 (5)	13 (6)	33
2010	De Socio							
HIV^+^		124 (11)	80 (9)	113 (11)	69 (9)	9 (6)	13 (8)	35
HIV^-^		124 (9)	79 (7)	109 (8)	67 (6)	12 (5)	17 (7)	15
2011	Bernardino							
HIV^+^		135 (14)^a^	88 (11)^a^	124 (16)^a^	77 (13)^a^	9 (6)	12 (8)	60
2012	Grandi^b^							
HIV^+^/hypertensive		141 (11)	93 (8)	124 (13)	76 (14)	NR	NR	NR
HIV^-^/hypertensive		140 (10)	93 (8)	124 (13)	75 (11)	NR	NR	NR
HIV^+^/normotensive		121 (9)	77 (7)	107 (8)	64 (7)	NR	NR	NR
HIV^-^/normotensive		123 (8)	75 (6)	108 (7)	63 (7)	NR	NR	NR
2013	Maggi							
HIV^+^		148 (8)^a^	91 (5)^a^	130 (10)^a^	79 (8)^a^	NR	NR	29
HIV^-^		149 (8)^a^	89 (4)^a^	129 (9)^a^	80 (7)^a^	NR	NR	21
2013	Schillaci							
HIV^+^		136 (16)	86 (11)	119 (16)	72 (11)	13 (9)	16 (9)	31
HIV^-^		133 (11)	86 (9)	115 (11)	70 (8)	13 (7)	19 (8)	16
2014	Borkum							
HIV^+^ (pre-ART)^c^		114 (4)	75 (12)	110 (6)	65 (8)	5 (6)^a^	11 (8)^a^	80
HIV^+^ (6 months ART)^c^		116 (12)	72 (11)	111 (4)	67 (11)	6 (5)^a^	12 (6)^a^	82
HIV^-^		114 (14)	73 (16)	99 (6)	60 (9)	10 (7)^a^	16 (8)^a^	53

Abbreviations: ART = Antiretroviral therapy; BP = Blood pressure; DBP = Diastolic blood pressure; NR = not reported; SBP = Systolic blood pressure; SD = Standard deviation

^a^Measures obtained through manuscript author correspondence. All author measures reported in the original manuscripts

^b^Grandi, 2012 HIV^-^ patients were matched to HIV^+^ patients by 24-hr SBP and DBP

^c^Borkum, 2014 were reported in 30 HIV^+^ individuals at baseline before initiating ART at baseline and subset of 28 HIV^+^ individuals adherent to ART for 6 months.

### Meta-Analyses comparing HIV^+^ and HIV^-^ Individuals

There were a total of 420 HIV^+^ and 714 HIV^-^ individuals from 7 studies. Meta-analyses for each BP outcome included individuals from 4–6 studies.

#### Mean BP values

**Fig 2:**
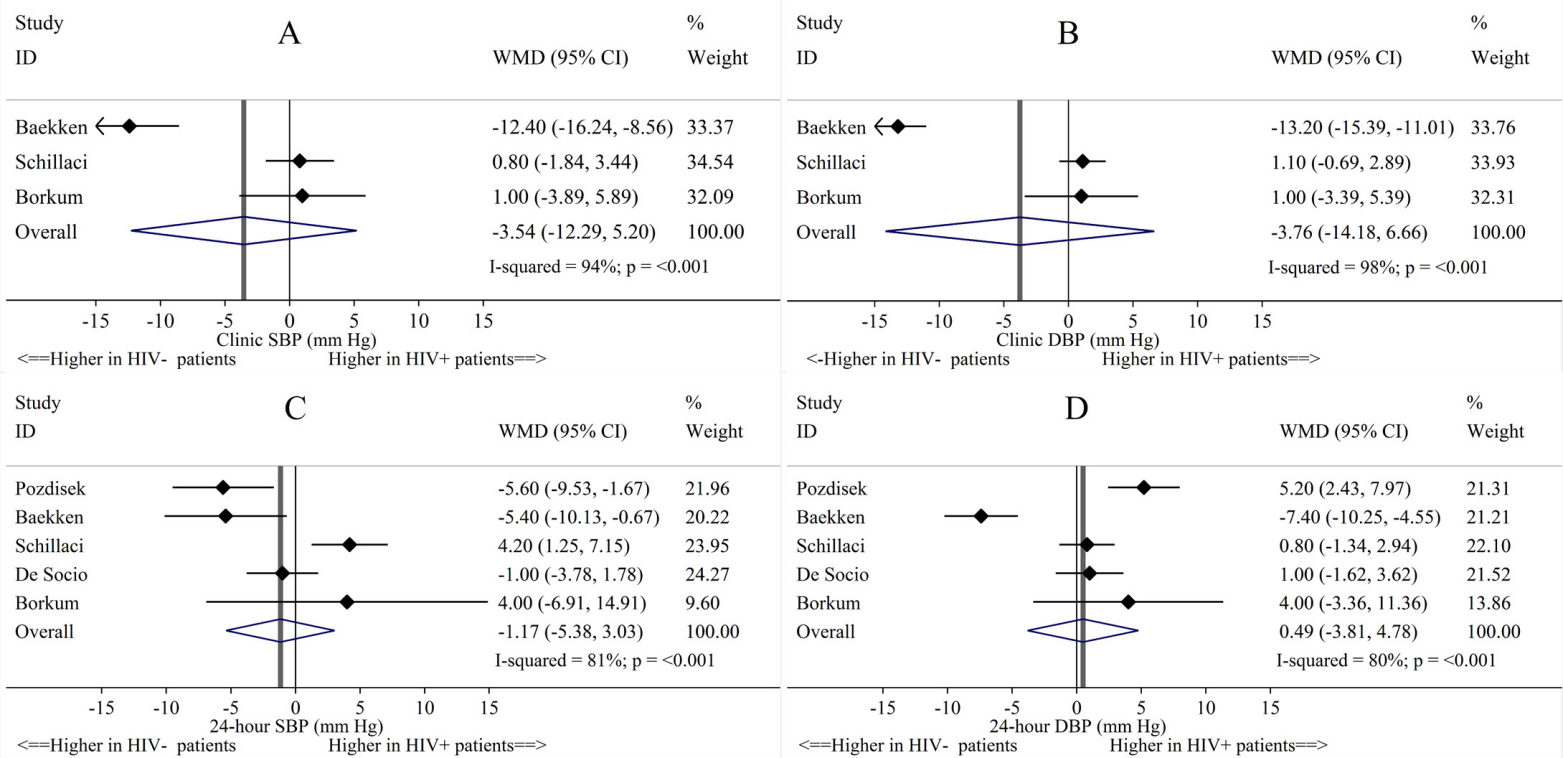
Study-specific and pooled mean differences of clinic and 24-hour blood pressure, in HIV^+^ compared to HIV^-^ study individuals. Abbreviations: CI = Confidence interval; DBP = Diastolic blood pressure; SBP = Systolic blood pressure; WMD = Weighted mean difference. Fig 2 shows the differences in (A) clinic SBP, (B) clinic DBP, (C) 24-hour SBP, and (D) 24-hour DBP between HIV^+^ and HIV^-^ individuals.

**Fig 3:**
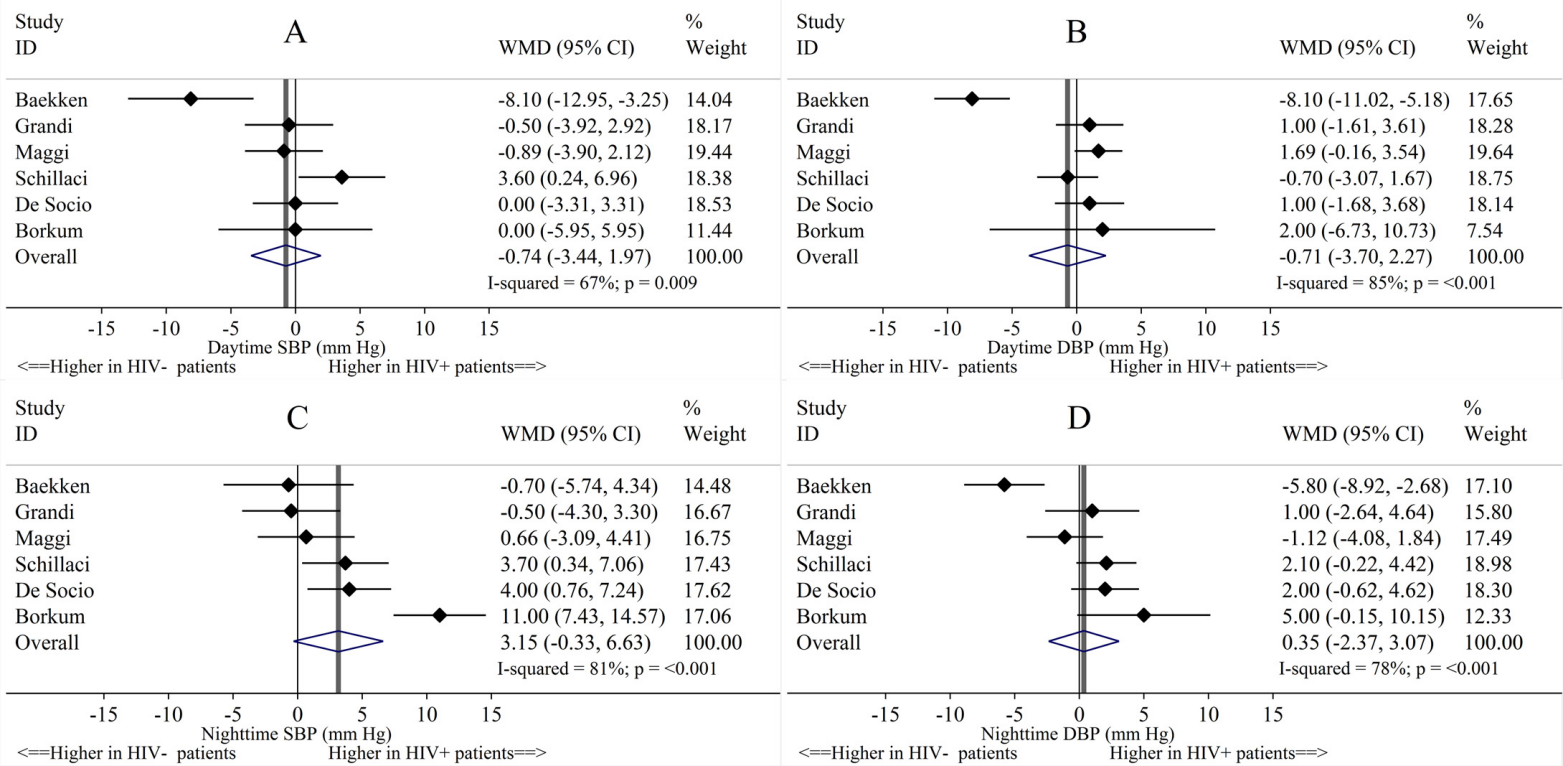
Study-specific and pooled mean differences of daytime and nighttime systolic and diastolic blood pressure, in HIV^+^ compared to HIV^-^ study individuals. Abbreviations: CI = Confidence interval; DBP = Diastolic blood pressure; SBP = Systolic blood pressure; WMD = Weighted mean difference. Fig 3 shows the differences in (A) daytime SBP, (B) daytime DBP, (C) nighttime SBP, and (D) nighttime DBP between HIV^+^ and HIV^-^ individuals.

Pooled clinic SBP and DBP were not statistically significantly different between HIV^+^ and HIV^-^ individuals in the 3 studies that reported these values and did not match on clinic BP or hypertension (Fig 2). Pooled mean 24-hour SBP and DBP were not statistically significantly different between HIV^+^ and HIV^-^ individuals in the 5 studies that reported these measures and did not match on 24-hour BP. Comparing HIV^+^ and HIV^-^ individuals, the pooled differences in mean daytime SBP and DBP and nighttime SBP and DBP were not statistically significantly different in the 6 studies that reported these measures (Fig 3).

### Nocturnal BP Decline and non-dipping BP

**Fig 4:**
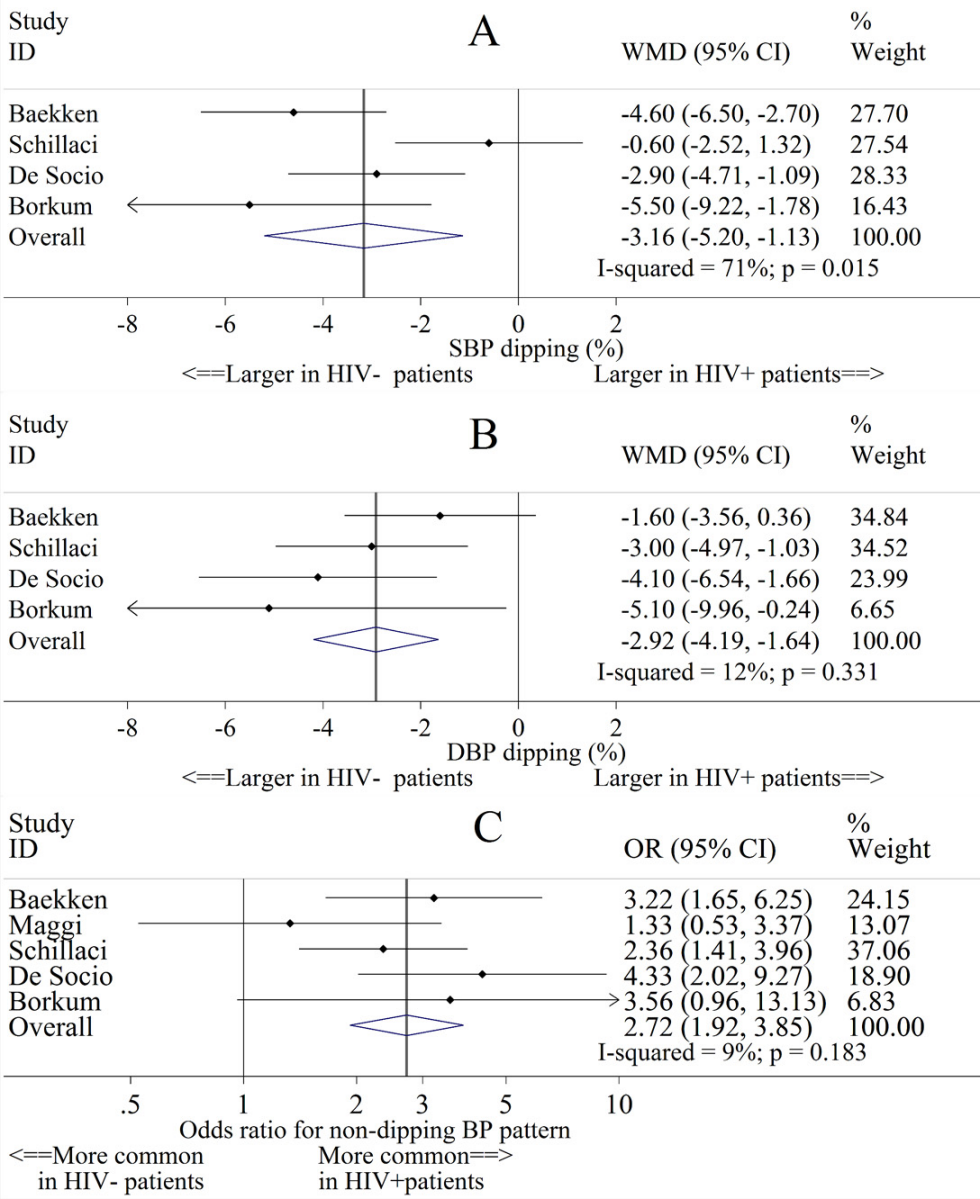
Study-specific and pooled mean differences (MD) of nocturnal blood pressure (BP) decline, and odds ratios (OR) of the presence of a non-dipping BP pattern, in HIV^+^ compared to HIV^-^ study individuals. Abbreviations: CI = Confidence interval; DBP = Diastolic blood pressure; OR = Odds ratio; SBP = Systolic blood pressure; WMD = Weighted mean difference. Fig 4 shows the absolute differences in (A) percent SBP dipping, (B) percent DBP dipping between HIV^+^ and HIV^-^ individuals, and (C) the odds ratio for non-dipping SBP for HIV^+^ versus HIV^-^ individuals.

Smaller nocturnal SBP and DBP declines were present in HIV^+^ compared with HIV^-^ individuals (Fig 4). Pooling data from 4 studies, the absolute differences in nocturnal SBP and DBP declines were 3.16% (95% CI: 1.13%, 5.20%) and 2.92% (95% CI: 1.64%, 4.19%) smaller, respectively among HIV^+^ compared with HIV^-^ individuals. In a pooled analysis of 5 studies, HIV^+^ individuals were more likely than their HIV^-^ counterparts to have a non-dipping SBP pattern (OR = 2.72 [95% CI: 1.92, 3.85]).

### Heterogeneity and sensitivity analyses

I^2^ statistics and heterogeneity chi-squared p-values indicated significant heterogeneity between study estimates for all BP outcomes except for nocturnal DBP decline and non-dipping SBP. Sensitivity analyses indicated that the exclusion of Baekken, 2009 or Schillaci, 2013 reduced the heterogeneity between study estimates and changed pooled estimates, depending on the outcome under study (Table 4). When Baekken, 2009 was removed from meta-analyses I^2^ statistics were reduced and heterogeneity chi-squared estimates changed from being significant to non-significant for clinic SBP and DBP and mean 24-hour SBP, daytime SBP and DBP, and nighttime DBP. Also, with Baekken, 2009 removed, all of these estimates moved towards being more positive (i.e., in the direction indicating HIV^+^ individuals had larger mean BP values); in particular, 24-hour DBP changed to having no significant difference by HIV status to being significantly higher in HIV^+^ individuals compared to HIV^-^ individuals, and nighttime SBP changed from having no significant difference by HIV status to being marginally significantly higher in HIV^+^ individuals compared to HIV^-^ individuals. When Schillaci, 2013 was removed from meta-analyses I^2^ statistics were reduced and heterogeneity chi-squared estimates changed from being significant to non-significant for mean 24-hour and daytime SBP. When Borkum, 2014 was removed, the mean nighttime SBP model results no longer had significant heterogeneity (Difference in HIV^+^ vs. HIV^-^ = 1.78 mm Hg [95% CI: -0.22, 3.78]; I^2^ = 30%; heterogeneity χ^2^ p = 0.224). The statistical significance of heterogeneity estimates did not change with the removal of other individual studies.

**Table 4:** Pooled estimates and heterogeneity statistics for meta-analyses of blood pressure measures, overall and excluding results from Baekken, 2009 and Schillaci, 2013.

	Including all studies			Excluding Baekken, 2009			Excluding Schillaci, 2013		
Outcome	Pooled association (95% CI)	I^2^	Heterogeneity χ^2^ p-value	Pooled association (95% CI)	I^2^	Heterogeneity χ^2^ p-value	Pooled association (95% CI)	I^2^	Heterogeneity χ^2^ p-value
Clinic SBP, mm Hg	-3.54 (-12.29, 5.20)	94%	<0.001	0.85 (-1.48, 3.17)	0%	0.944	-5.79 (-18.91, 7.34)	94%	<0.001
Clinic DBP, mm Hg	-3.76 (-14.18, 6.66)	98%	<0.001	1.09 (-0.57, 2.75)	0%	0.967	-6.23 (-20.15, 7.68)	97%	<0.001
24-hour SBP, mm Hg	-1.17 (-5.38, 3.03)	81%	<0.001	-0.10 (-4.80, 4.59)	82%	0.001	-3.08 (-6.36, 0.203)	52%	0.101
24-hour DBP, mm Hg	0.49 (-3.81, 4.78)	80%	<0.001	2.38 (0.01, 4.74)	58%	0.067	0.49 (-5.61, 6.59)	93%	<0.001
Daytime SBP, mm Hg	-0.74 (-3.44, 1.97)	67%	0.009	0.45 (-1.24, 2.14)	12%	0.339	-1.64 (-4.20, 0.91)	53%	0.072
Daytime DBP, mm Hg	-0.71 (-3.70, 2.27)	85%	<0.001	0.88 (-0.26, 2.03)	0%	0.640	-0.70 (-4.51, 3.11)	88%	<0.001
Nighttime SBP, mm Hg	3.15 (-0.33, 6.63)	81%	<0.001	3.80 (0.00, 7.61)	83%	<0.001	3.01 (-1.35, 7.36)	85%	<0.001
Nighttime DBP, mm Hg	0.35 (-2.37, 3.07)	78%	<0.001	1.47 (-0.11, 3.05)	24%	0.262	-0.02 (-3.35, 3.30)	80%	0.001
Nocturnal SBP decline, %	-3.16 (-5.20, -1.13)	71%	0.015	-2.61 (-4.99, -0.24)	68%	0.044	-3.95 (-5.35, -2.56)	17%	0.301
Nocturnal DBP decline, %	-2.92 (-4.19, -1.64)	12%	0.331	-3.58 (-5.04, -2.13)	0%	0.642	-3.07 (-5.15, -1.00)	41%	0.183
Non-dipping SBP pattern, OR	2.72 (1.92, 3.85)	9%	0.183	2.59 (1.63, 4.12)	26%	0.253	2.93 (1.79, 4.79)	24%	0.268

DBP = Diastolic blood pressure; OR = Odds ratio; SBP = Systolic blood pressure.

## Discussion

In this systematic review and meta-analysis, we identified 8 studies that reported ABPM measures in cohorts of HIV^+^ individuals [20–27]. While study methods and populations differed, 7 of these 8 studies were European and no study participants were taking antihypertensive medication. Seven studies included HIV^-^ individuals as a comparison group and each BP outcome examined in the current meta-analysis was reported in 4–6 studies. When these studies were pooled, the differences between HIV^+^ and HIV^-^ individuals in clinic SBP and DBP, and ABPM-measured mean 24-hour, daytime, and nighttime SBP and DBP were not statistically significant. Individual study estimates for these outcomes were highly heterogeneous and some results differed when individual studies were removed from meta-analyses. However, less nocturnal SBP and DBP declines and a higher prevalence of non-dipping SBP, measures and phenotypes associated with an increased risk for CVD in the general population, were consistently reported in HIV^+^ compared to HIV^-^ individuals [11]. Individual study estimates for differences in nocturnal DBP decline and non-dipping BP did not indicate a high degree of heterogeneity, but individual estimates for differences in nocturnal SBP did indicate heterogeneity. When Schillaci, 2013 was removed from the nocturnal SBP decline meta-analysis there was no longer an indication for heterogeneity, and a smaller nocturnal SBP decline among HIV^+^ individuals remained. Only 2 studies reported the prevalence of white coat hypertension, each defined it differently, and neither reported this measure in HIV^-^ controls [18, 27]. No studies reported 24-hour BP variability, or the prevalence of non-dipping DBP or masked hypertension among HIV^+^ individuals.

The differences in dipping BP by HIV status suggested by this meta-analysis may occur due the HIV infection itself or through the psychosocial impact of living with HIV. HIV-induced proteins in mouse models have been shown to alter the body’s circadian clock in a similar manner as light [9]. Also, monkeys with simian immunodeficiency virus (SIV) have altered diurnal body temperature and physical activity [9]. Autonomic dysfunction and abnormal baroreflex function have been reported in HIV^+^ individuals [5]. Decreased heart rate variability and other markers of autonomic dysfunction are associated with an increased risk of CVD and an increased likelihood of a non-dipping SBP pattern and hypertension based on mean 24-hour BP, independent of hypertension based on clinic BP [6, 7, 28, 29]. HIV infection induces a chronic inflammatory state that likely contributes to excess CVD risk in this population [1, 30]. Similarly, inflammatory biomarkers have been reported to be higher in individuals with a non-dipping SBP pattern [31]. While effective ART can partially restore the immune function and attenuate inflammation, treated HIV^+^ individuals still have higher levels of inflammatory biomarkers and elevated CVD risk [1, 3, 30]. In the current systematic review, individual studies consisting of HIV^+^ patient samples that were over 80% or 0% on ART reported that a non-dipping SBP pattern was more common among those with HIV^+^ versus HIV^-^. In addition, one single-arm trial found in the review reported that the prevalence of a non-dipping SBP pattern in HIV^+^ individuals was 80% and 82% before and after 6 months of ART, respectively, compared to 53% in HIV^-^ controls [21]. However, data are limited and we cannot exclude the possibility that ART has some impact on ABPM measures. Also, factors other than HIV infection and ART may affect ABPM measures in HIV^+^ individuals. HIV^+^ status is associated with higher psychological stress levels, social stigma, pain and sleep disturbances, which may affect nocturnal BP decline, particularly among HIV^+^ racial/ethnic minorities and other groups that experience additional stigmas and poorer sleep quality [8–10, 32].

Reduced BP declines may explain part of the increased risk for CVD present in HIV^+^ individuals [1, 15, 33]. In a meta-analysis of 3 studies, HIV^+^ individuals had 1.61 (95% CI: 1.43, 1.83) times the risk for CVD outcomes compared with their HIV^-^ counterparts [3]. An increased CVD risk associated with HIV^+^ status has been reported even after adjustment for CVD risk factors, including hypertension based on clinic BP and antihypertensive treatment [1]. Studies in the general population have also demonstrated that a non-dipping SBP pattern is associated with higher CVD and mortality risk, after accounting for treatment and BP [34–36]. For example, a study performed in a sample with untreated isolated systolic hypertension reported that each 10% absolute higher nighttime-to-daytime SBP ratio was associated with a hazard ratio for CVD events of 1.41 (95% CI: 1.03, 1.94), even after adjustment for 24-hour mean SBP [34]. Another study reported that a non-dipping SBP pattern was associated with a hazard ratio for CVD mortality of 2.50 (97% CI: 1.28, 4.88) [37]. A non-dipping SBP pattern has also been associated with an increased risk for congestive heart failure and non-cardiovascular death in the general population [35, 36].

It has been suggested that individuals with reduced nocturnal BP decline may benefit from taking antihypertensive medication at night, rather than in the morning [38]. In a randomized controlled trial of 2,156 individuals from the general population, taking antihypertensive medication at night versus during the day was associated with a significantly larger nocturnal SBP and DBP dip and a reduction in CVD events [39]. This trial reported that after adjustment for mean BP on ABPM, each 5% absolute larger nocturnal SBP and DBP dipping was associated with hazard ratios of 0.87 (95% CI: 0.81, 0.94) and 0.86 (95% CI: 0.80, 0.92), respectively, for CVD [39]. However, this study included only ethnically homogeneous participants in a single Spanish center, and utilized a 48-hour ABPM period rather than the usual 24-hour period [40]. In addition, a study in African Americans with kidney disease reported that taking antihypertensive medication at night, compared with taking medication in the morning, did not lower nighttime BP [41]. These and future studies of the associations between timing of antihypertensive medication and non-dipping BP and CVD outcomes may have particular relevance to HIV^+^ individuals given their high prevalence of non-dipping SBP.

Another potential, but understudied approach to increase nocturnal BP declines in HIV^+^ individuals may be to address the high prevalence of sleep disorders and psychosocial burden present in this population, which may adversely affect BP dipping [9, 32, 42–50]. Interventions addressing psychosocial factors are important to improve the quality of life and care in HIV^+^ individuals, but there are few data on these interventions would affect nocturnal BP declines. Treatment for sleep apnea has been shown to reduce nighttime blood pressures in the general population and may be useful for HIV^+^ individuals with non-dipping SBP [51, 52]. There are few other data on whether the treatment of other forms of sleep disturbances and poor sleep quality (e.g., insomnia) have an effect on nocturnal BP decline or nighttime BP [53]. Future research should examine whether mental health or sleep-related interventions can increase nocturnal BP decline and decrease CVD risk in HIV^+^ individuals.

All but one study was conducted in predominantly white European populations, with the remaining study in black South Africans. We did not find any studies that reported ABPM phenotypes in African Americans with HIV. In the US, African Americans are disproportionately affected by HIV [54]. Both in the general population and among people with HIV, African Americans have higher clinic BP and have an increased risk for BP-related outcomes compared with whites [55–58]. In a study of a general US population sample participating in the Coronary Artery Risk Development in Young Adults (CARDIA) study, the prevalence of a non-dipping SBP pattern was 48% and 27% in young (mean age 30 years) African Americans and whites, respectively [57]. Furthermore, African Americans were more likely than whites to have masked hypertension, and had higher levels of BP variability [57]. Future studies are needed to determine whether similar racial differences exist between African American and white HIV^+^ individuals in the US.

The current systematic review and meta-analyses should be interpreted in the context of known and potential limitations. The designs of the identified studies varied substantially. For example, some studies matched HIV^+^ and HIV^-^ individuals on clinic or mean 24-hour BP, whereas others did not. This may have resulted in the heterogeneous estimates seen in this analysis. Additionally, since not all BP measures were reported in every study, meta-analyses for different BP outcomes were performed among different sets of studies. Specifically, meta-analyses examining differences in nocturnal SBP and DBP declines contained 4 studies, meta-analyses examining differences in non-dipping SBP pattern contained these 4 studies and an additional 5th study, and meta-analyses examining differences in daytime and nighttime BP by HIV status contained an additional 6th study. Having different sets of included studies by BP outcome may be a contributing reason that the current analysis found differences in nocturnal BP declines and SBP dipping by HIV status despite not finding differences in either daytime or nighttime BP by HIV status. A separate limitation of the current meta-analyses is that we could not examine whether differences in ABPM measures by HIV-status were due to the viral infection, psychosocial factors, or sleep disturbances and quality. Finally, none of the studies included the assessment of associations between ABPM measures and CVD outcomes.

In conclusion, in the current meta-analysis, HIV^+^ individuals had less nocturnal declines in SBP and DBP and were more likely to have a non-dipping SBP pattern compared to their HIV^-^ counterparts. These associations were present despite similar mean levels of clinic, 24-hour, daytime, and nighttime BP across HIV status. Only two studies reported white coat hypertension in HIV^+^ individuals and no studies reported the prevalence of masked hypertension or 24-hour BP variability among HIV^+^ individuals. The value of using ABPM to guide antihypertensive treatment in the general population is becoming more widely recognized, but is still not being extensively utilized in clinical practice and few data are available to guide its use in individuals with HIV [11, 16]. Larger studies of ABPM in HIV^+^ individuals are needed to more reliably estimate the prevalence of ABPM phenotypes in this population and determine whether they contribute to increased CVD risk.

## Supporting Information

S1 Appendix Search terms used for the Ovid MEDLINE search in the systematic literature review of ambulatory blood pressure measures in HIV^+^ individuals. (DOCX)

S1 Table Additional blood pressure data included in studies of HIV^+^ individuals. (DOCX)
