# Supplementary material for: Ambulatory Blood Pressure Monitoring in Individuals with HIV: A Systematic Review and Meta-Analysis
Source: PLoS One. 2016 Feb 16;11(2):e0148920. doi: 10.1371/journal.pone.0148920 (PMC4755611; doi:10.1371/journal.pone.0148920)
Supplement: S1 Appendix — (DOCX) [file pone.0148920.s001.docx]

S1 Appendix. Search terms used for the Ovid MEDLINE search in the systematic literature review of ambulatory blood pressure measures in HIV^+^ individuals

1. exp HIV/

2. hiv.tw.

3. human immunodeficiency virus$.tw.

4. exp HIV Infections/

5. aids.tw.

6. Acquired Immunodeficiency Syndrome.tw.

7. or/1-6

8. Blood Pressure/

9. Blood Pressure Monitoring, Ambulatory/

10. (abp or abpm).tw.

11. ((ambulatory or day$ or diurnal or "24" or twenty four) adj2 (bp or blood pressure)).tw.

12. ((nocturnal or masked or white coat) adj hypertens$).tw.

13. non-dipping.tw.

14. or/8-13

15. 7 and 14

16. limit 15 to humans
