# Supplementary material for: Ambulatory Blood Pressure Monitoring in Individuals with HIV: A Systematic Review and Meta-Analysis
Source: PLoS One. 2016 Feb 16;11(2):e0148920. doi: 10.1371/journal.pone.0148920 (PMC4755611; doi:10.1371/journal.pone.0148920)
Supplement: S1 Table — (DOCX) [file pone.0148920.s002.docx]

S1 Table. Additional blood pressure data included in studies of HIV^+^ individuals

| Blood pressure measure and study | HIV^+^ | HIV^-^ |
| --- | --- | --- |
| 24-hour hypertension, % |  |  |
| Pozdisek, 2008 | 35% | 35% |
| Daytime hypertension, % |  |  |
| Manner, 2010* | 74% | - |
| Nighttime hypertension, % |  |  |
| Manner, 2010* | 81% | - |
| Rising nighttime BP pattern, % |  |  |
| Bernardino, 2011 | 12% | - |
| Extreme nighttime dipping BP pattern, % |  |  |
| Bernardino, 2011 | 5% | - |
| Clinic pulse pressure, mean mm Hg (SD) |  |  |
| Schillaci, 2013 | 51 (10) | 51 (10) |
| 24-hour pulse pressure, mean mm Hg (SD) |  |  |
| Schillaci, 2013 | 49 (9) | 46 (7) |
| Daytime pulse pressure, mean mm Hg (SD) |  |  |
| Schillaci, 2013 | 51 (11) | 46 (8) |
| Nighttime pulse pressure, mean mm Hg (SD) |  |  |
| Schillaci, 2013 | 46 (9) | 45 (7) |
| Nocturnal mean arterial pressure percent decline, mean (SD) |  |  |
| Schillaci, 2013 | 14 (3) | 16 (7) |
| Ambulatory arterial stiffness index, mean (SD) |  |  |
| Schillaci, 2013 | 0·46 (0·22) | 0·29 (0·17) |
| Symmetric ambulatory arterial stiffness index, mean (SD) |  |  |
| Schillaci, 2013 | 0·22 (0·18) | 0·11 (0·15) |
| White coat hypertension, % |  |  |
| Manner, 2010^*†^ | 26% | - |
| Bernardino, 2011^††^ | 40% | - |

Abbreviations: BP = Blood pressure; HIV = Human immunodeficiency virus; SD = Standard deviation

Measures were obtained from the original manuscript.

^*^Manner, 2010 consisted of the 77 HIV+ individuals in in the Microalbuminuria in the HIV-infected population of OSLO (MAHO) cohort included in Baekken, 2009.

^†^Manner, 2010 calculated the prevalence of white coat hypertension as the proportion having daytime SBP <130 mm Hg and DBP < 85 mm Hg among those with clinic SBP ≥ 140 mm Hg or DBP 90 mm Hg.

^††^Bernardino, 2010 calculated the prevalence of white coat hypertension as the proportion having daytime BP as SBP <135 mm Hg and DBP < 85 mm Hg among those with clinic SBP ≥ 140 mm Hg or DBP 90 mm Hg.
